# Narrative Review of Hydration and Selected Health Outcomes in the General Population

**DOI:** 10.3390/nu11010070

**Published:** 2019-01-01

**Authors:** DeAnn Liska, Eunice Mah, Tristin Brisbois, Pamela L. Barrios, Lindsay B. Baker, Lawrence L. Spriet

**Affiliations:** 1 Biofortis, Mérieux NutriSciences, Addison, IL 60101, USA; eunice.mah@mxns.com; 2 PepsiCo, Inc., Purchase, NY 10577, USA; Tristin.Brisbois@pepsico.com (T.B.); Pamela.Barrios@pepsico.com (P.L.B.); 3 Gatorade Sports Science Institute, Barrington, IL 60010, USA; Lindsay.Baker@pepsico.com; 4 Department of Human Health and Nutritional Sciences, University of Guelph, Guelph, ON N1G 2W, Canada; lspriet@uoguelph.ca

**Keywords:** fluid, water, dehydration, skin, constipation, kidney, cognition, mood, headache, body weight, systematic review

## Abstract

Although adequate hydration is essential for health, little attention has been paid to the effects of hydration among the generally healthy population. This narrative review presents the state of the science on the role of hydration in health in the general population, specifically in skin health, neurological function (i.e., cognition, mood, and headache), gastrointestinal and renal functions, and body weight and composition. There is a growing body of evidence that supports the importance of adequate hydration in maintaining proper health, especially with regard to cognition, kidney stone risk, and weight management. However, the evidence is largely associative and lacks consistency, and the number of randomized trials is limited. Additionally, there are major gaps in knowledge related to health outcomes due to small variations in hydration status, the influence of sex and sex hormones, and age, especially in older adults and children.

## 1. Introduction

Water is essential for life and is involved in virtually all functions of the human body [1]. It is important in thermoregulation, as a solvent for biochemical reactions, for maintenance of vascular volume, and as the transport medium for providing nutrients within and removal of waste from the body [2]. Deficits in body water can compromise our health if they lead to substantial perturbations in body water balance [2]. As with other essential substances, intake recommendations for water are available from various authoritative bodies [e.g., Institute of Medicine (IOM) and European Food Safety Authority (EFSA)], and generally range from 2–2.7 L/day for adult females and 2.5–3.7 L/day for adult males [1,2].

Body water balance depends on the net difference between water gain and water loss. The process of maintaining water balance is described as “hydration”. “Euhydration” defines a normal and narrow fluctuation in body water content, while “hypohydration” and “hyperhydration” refer to a generalized body water deficit or excess, respectively, beyond the normal range. Finally, “dehydration” describes the process of losing body water while “rehydration” describes the process of gaining body water. Dehydration can be further classified based on the route of water loss and the amount of osmolytes (electrolytes) lost in association with the water. Iso-osmotic hypovolemia is the loss of water and osmolytes in equal proportions, which is typically caused by fluid losses induced by cold, altitude, diuretics, and secretory diarrhea. Hyperosmotic hypovolemia occurs when the loss of water is greater than that of osmolytes, and primarily results from insufficient fluid intake to offset normal daily fluid losses (e.g., loss of pure water by respiration and transcutaneous evaporation). Hyperosmotic hypovolemia is exacerbated with high sweat loss (warm weather or exercise) or osmotic diarrhea [3,4].

The normal daily variation of body water is <2% body mass loss (~3% of total body water); thus, hypohydration is clinically defined as ≥2% body mass deficit [5]. The kidneys can regulate plasma osmolality within a narrow limit (±2% or 280 to 290 mOsm/kg) and plasma osmolality between 295 and 300 mOsm/kg is considered mild or impending hyperosmotic hypovolemia, while values greater than 300 mOsm/kg are considered frank hyperosmotic hypovolemia [6,7]. For urine osmolality, values above 1000 mOsm/L are considered elevated and may be a sign of hyperosmotic hypovolemia [6,7]. Finally, it has generally been accepted that a first-morning void urine specific gravity (USG) of less than or equal to 1.020 represents euhydration [7,8].

Hydration status is assessed in a variety of ways in human studies, the most common of which are body weight changes, plasma and/or urine osmolality, and USG. The choice of hydration assessment method and its interpretation is dependent on the type of dehydration; for example, iso-osmotic hypovolemia does not increase plasma or serum osmolality and USG due to the concurrent loss of salt and water. Further complicating the assessment of hydration status are confounding factors such as age and differences in renal function, and these limitations among others have been covered in detail elsewhere [6,9,10,11,12,13,14,15,16]. In addition to assessing hydration status, studies investigating the effects of hydration on health often include measurements of fluid intake, which is usually conducted with dietary assessment methods such as dietary record, diet recall, or food frequency questionnaires. A full assessment of the advantages and limitations of these methods (e.g., difficulties with method validation, challenges in usage among children and the elderly with cognitive issues) are outside the scope of the current review, but have been discussed in detail by others [17,18].

Several reviews on the role of hydration in disease development and progression, as well as the role of hydration in exercise and physical performance have been published [19,20,21,22,23]. However, few reviews are available on the role of hydration in general health, with the exception of a few outcome areas (e.g., weight loss, cognition). An assessment of the role of hydration in general health that thoroughly evaluates the evidence related to the commonly believed benefits of hydration is not available. Thus, the objective of this review was to provide an assessment of the current state of science on hydration and health relevant to the general population. This review includes skin health, neurological, gastrointestinal and renal functions, and body weight and composition in relation to hydration in generally healthy individuals. Publications reviewed include the most current systematic reviews and meta-analyses as well as primary intervention studies published since these reviews.

## 2. Materials and Methods

The PubMed database was initially searched for reviews on hydration that were published in English. All searches and screening were performed independently by two authors. Reviews were identified using the search terms “hydration” and “dehydration” and selection included those that were conducted using a systematic search process and related to a health area applicable to the general population. In the absence of systematic reviews and meta-analyses, comprehensive narrative reviews that included details on the studies reviewed were included, while opinion pieces were excluded. In addition, hand-searching of references in selected reviews were performed. Key systematic and comprehensive reviews and meta-analyses are summarized in Table 1. 

In order to represent the current state of the literature, primary studies that were not included in the reviews were also identified. Individual searches for clinical intervention trials in generally healthy populations (ages >2 years) and excluding those conducted in diseases populations were conducted in PubMed using the All Fields (ALL) function for terms for hydration and the specific health outcome area. When a systematic review had been identified, the updated search for primary literature overlapped the search in the published systematic review by a year. When a systematic review of a specific topic was not found, the search for primary literature was performed in PubMed starting from its inception. Search terms were compared with the systematic reviews on each topic, when available.

**Figure 1:**
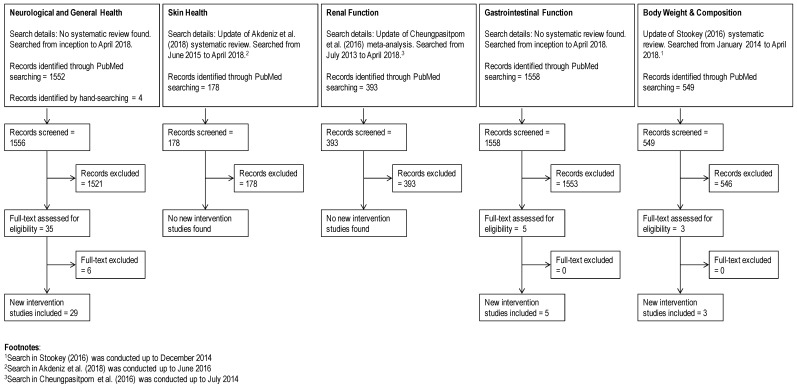
PRISMA flowchart.

A flowchart documenting the updated search strategy and results is shown in Figure 1. The updated search for weight management used the terms “(fluid OR water OR hydration OR dehydration) AND (weight OR BMI (body mass index) OR circumference)”. For hydration and skin, the terms included “(fluid OR water OR hydration OR dehydration) AND (skin OR epidermal OR transepidermal) NOT (topical OR injection OR injector)”. Search terms for studies on hydration and neurological function were “(water OR hydration OR dehydration) AND (mental OR mood OR cognition OR fatigue OR sleep OR headache)” and studies in diseased population such as dementia were excluded. For gastrointestinal function, the search terms included “(fluid OR water OR hydration OR dehydration) AND (intestinal OR gastric OR constipation) NOT (infant OR cancer)”. Finally, for hydration and renal function, “(fluid OR water OR hydration OR dehydration) AND (kidney OR renal) NOT (infant OR cancer)” was used and studies involving diseased populations such as chronic kidney disease were excluded. Only clinical trials that were not included in systematic reviews are reported in detail in each health outcome section. Information from each primary study was extracted using a pre-determined PICOS (population, intervention, comparison, outcome, study design) table, making sure that any reports of hydration status (e.g., body weight change, plasma osmolality) or fluid intake were recorded.

Finally, the reporting and methodological qualities of each systematic review and meta-analysis was assessed using the Preferred Reporting Items for Systematic Reviews and Meta-Analyses (PRISMA) checklist (http://www.prisma-statement.org/) and A MeaSurement Tool to Assess systematic Reviews (AMSTAR) 2 (https://amstar.ca/index.php). For meta-analyses, the PRISMA checklist contained 24 required reporting items and three optional items [item 16 (description of additional analyses, if performed), item 19 (reporting of data on risk of bias for each study, if performed), and item 23 (reporting of results of additional analysis, if performed)]. Only the required items were used for scoring. For systematic reviews, 19 items remained after exclusion of optional items and items specific to meta-analyses (i.e., items 13, 14, 15, 21, and 22, which are related to data analysis and risk bias assessment). AMSTAR 2 has 16 items in total, whereby three of these are specific for meta-analysis. 

## 3. Hydration and Health Outcomes

### 3.1. Skin Health

The skin’s primary functions are to protect the body from external challenges (e.g., chemicals, microbiological materials, and physical stressors), regulate water loss and body temperature, and sense the external environment [30,31,32]. The skin also serves as a reservoir for nutrients and water and contributes to important metabolic activities [30]. The external layer of the skin provides an epidermal barrier, which is composed of 15–20 layers of cornified keratinocytes (corneocytes). The stratum corneum (SC) layer of the epidermis is the primary location of the barrier function; however, both the dermis and the multilayered epidermis are important for maintenance of barrier integrity [32]. Measurements for skin barrier function and hydration include transepidermal water loss (TEWL), SC hydration, “deep” skin hydration, clinical evaluation of dryness, roughness and elasticity, skin relief parameter, the average roughness, evaluation of skin surface morphology, skin smoothness and roughness, extensibility, sebum content, and skin surface pH [24].

For hydration and skin health, a 2018 systematic review was identified [24], which included five intervention studies. Of these studies, four measured surface hydration and reported increased SC hydration following additional intake of 2 L daily of water over a period of 30 days [33,34,35] or additional intake of 1 L per day for a period of 42 days [36]. Of note, only one of these studies [34] compared the effect of additional water consumption in those who habitually consumed below or above the EFSA water requirement (2 L/day). Other studies either assessed participants who were habitually meeting or exceeding the European Food Safety Authority (EFSA) requirements [33,35] or failed to report baseline fluid intake [36,37]. In studies that stratified based on baseline water intake [33,34,35], positive impact on skin hydration was evident in participants whose baseline total water intake was less than 3.2 L per day. Measurements of dryness and roughness were reported in one study [36] and these decreased with additional water intake. Measurements of skin elasticity [36], extensibility [33], and the ability of the skin to return to its original state [35] were greater with additional water intake. However, the review authors concluded that the evidence is weak in terms of quantity and methodological quality, and risk of bias in the interventional studies is extremely high [24]. With the exception of providing an explicit statement of questions being addressed and clarifying if a review protocol existed, the 2018 systematic review fulfilled all required PRISMA reporting items (Table 1). The systematic review fulfilled only four out of the 13 required AMSTAR 2 items and lacked clarity on inclusion criteria and study selection, robustness of study selection, and completeness in description and assessment of included studies.

Our updated search resulted in 178 titles (Figure 1), but the vast majority assessed topical applications (e.g., moisturizers), oral ingestion of supplements or herbals, or skin conditions in disease states. No new intervention studies were found when compared with the 2018 systematic review [24].

### 3.2. Neurological Function

Studies on hydration and neurological function focused on cognition, mood, fatigue, sleep, and headache. In general, the areas of cognition, mood, and fatigue overlap in studies, with some also including sleep and headache outcomes. No single systematic review covered these various topics; however, comprehensive narrative reviews that included discussions on cognition [26] and headache [19] were identified. Thus, our search for primary literature on this topic was not limited to recent literature (Figure 1). After screening, 29 studies were selected and these are summarized in Table 2. Of the cognition studies, eight investigated children and adolescents, 18 adults, and one both children and adults, while two other studies looked at headaches in adults. None of the intervention studies were specific to fatigue or sleep.

#### 3.2.1. Cognition, Mood, and Fatigue in Adults

Cognition is a complex function that is composed of several subdomains including different types of memory, attentiveness, reaction time, and executive function. Studies often differ in the specific subdomains assessed as well as the tool used for these measurements. Assessments of mood are also varied across studies, with a number of different types of questionnaires; although a consensus approach does not exist, some validated questionnaires are available [e.g., Bond-Lader, Profile of Moods States (POMS)] and these are the most commonly used. 

Several recent reviews of the data in adults have been published (Table 1). Benton and Young [25] concluded that reductions in body mass by >2% due to dehydration are consistently associated with greater fatigue and lower alertness; however, the effects on cognition is less consistent. Masento et al. [26] summarized that severe hypohydration was shown to have detrimental effects on short-term memory and visual perceptual abilities, whereas water consumption can improve cognitive performance, particularly visual attention and mood. These authors also note some of the challenges with studying hydration effects on mood and cognition include variations among subjects (e.g., differing levels of thirst at baseline, habitual intake, and individual adaptation), mediating factors (e.g., water temperature, time of cognition testing, testing environment), variation in types of cognition tests, and distinguishing effects due to thirst and hydration status.

As noted by previous review authors, the studies we identified on attention are heterogeneous in methodology and outcomes. Studies included acute and chronic water consumption, with or without initial dehydration, and measured various types of attention including visual, sustained, and focused (Table 2). Following an overnight fast, acute intake of 25, 200, or 300 mL water improved visual attention [50,38]. Acute intake of water (120 or 330 mL) immediately before testing improved sustained attention in one study [62], but not in another that required overnight fasting prior to consumption of 300 mL water [61]. Both studies assessed sustained attention using the Rapid Visual Information Processing task although the former allowed habitual fluid intake prior to cognition assessments (performed at 11 am or 3 pm), and the latter restricted fluid and food intake for 9 h prior to testing. When dehydration was induced by exercise with or without diuretic (body mass loss of ≥1%), sustained attention decreased compared to exercise with fluid replacement in men [56]. However, a similar dehydration and euhydration protocol did not affect sustained attention in women [55]. Dehydration induced by water deprivation (average body mass loss of 1%) also did not affect visual attention [47]. Under hot conditions (30 °C), dehydration (mean body mass loss of 0.7%) followed by water consumption improved focused attention compared to dehydration without water consumption [48]. Fluid restriction for 28 h (mean body mass loss of 2.5%) did not affect visual, sustained, and divided attention, although subjects reported needing a greater amount of effort and concentration necessary for successful task performance when dehydrated compared to euhydration [59]. Finally, in dose–response studies, attention deteriorated starting at 2% body mass loss [63,64].

Studies on memory are equally heterogeneous in methodology and results. Working memory has been shown to improve following acute intake of water by some [38], but not others [50,61]. When dehydration was induced by exercise with or without diuretic (body mass loss of ≥1%), working memory decreased compared to exercise with fluid replacement in men [56]. However, a similar dehydration and euhydration protocol did not affect working and short-term memory in women [55]. Dehydration following water deprivation (average body mass loss of 1%) increased errors for tests for visual/working memory [47]. Working memory was also unaffected by dehydration (body mass loss of 1 to 3%) induced by two weeks of low-fluid diet (≤40 oz fluid/day or ≤1.2 L/day) [51]. Additionally, more extreme hypohydration (mean body mass loss of 4%) did not affect short-term spatial memory in men [53]. Under hot conditions (30 °C), dehydration (mean body mass loss of 0.7%) followed by water consumption improved episodic memory compared to dehydration without water consumption [48]. Finally, in dose–response studies, short-term memory started deteriorating after 2% body mass loss [63,64]. 

Compared with attention and memory, fewer studies assessed reaction time. Simple reaction time was unaffected by acute consumption of 200 mL water prior to testing [50]. In another study that evaluated thirst sensation, subjects who were thirsty and provided 0.5–1 L of water had better simple reaction time compared to thirsty subjects who did not consume water [52]. Choice reaction time was unaffected by hypohydration in women (≥1% body mass loss) [55] or in men (mean body mass loss of 4%) [53]. Both simple and choice reaction time were unaffected by hypohydration in women (average body mass loss of 1%) [47]. 

Other lesser studied cognitive subdomains include grammatical reasoning, spatial cognition, verbal response time, and executive function. Grammatical reasoning was unaffected by hypohydration in women (≥1% body mass loss) [55] or in men (mean body mass loss of 4%) [53]. Flight performance and spatial cognition of healthy pilots were compromised by dehydration (body mass loss of 1 to 3%) induced by 2 weeks of low-fluid diet (≤40 oz fluid/day or ≤1.2 L/day) [51]. Hypohydration following 28 h of fluid restriction (mean body mass loss of 2.5%) decreased verbal response time in women, but increased verbal response time in men and did not affect cognitive-motor speed in either women or men [59]. Smaller degree of dehydration by fluid restriction (mean body mass loss of 1.08%) also did not affect motor speed and visual motor function, visual learning, and cognitive flexibility, but decreased executive function and spatial problem solving [47]. Speed, accuracy, and mental endurance were decreased after 3 h of fluid deprivation (500 g fluid/day), and decreased stability occurred after 35 h [58]. Finally, in dose–response studies, arithmetic efficiency, motor speed, and perceptual motor coordination deteriorated starting at 2% body mass loss [63,64].

Overall, negative emotions such as anger, hostility, confusion, depression, and tension as well as fatigue and tiredness increase with dehydration of ≥1% [53,55,56,59,60] and fluid deprivation (24 h [54]). In men, fluid deprivation (500 g (or ~500 mL) fluid for 24 h) decreased energy ratings after 15 h but did not affect depression, anxiety, and self-confidence [58]. Only one study assessed water consumption following dehydration and demonstrated decreased anxiety, but not depression, when mildly dehydrated subjects (mean body mass loss of 0.2%) were provided with water [48]. Acute water intake by subjects after an overnight fast did not affect various mood ratings [50,61]. Additionally, although thirsty subjects were more tired and tense, provision of 0.5–1 L water did not affect tired and tense ratings [52]. It is possible that mood effects of acute water consumption in these studies were not observed due to the timing of testing relative to water consumption (often >20 min). Indeed, acute water intake (120 and 220 mL) increased alertness assessed after 2 min, but not when assessed after 25 or 50 min of water consumption [62]. Increasing water intake of low-consumers (<1.2 L/day) decreased confusion/bewilderment scores and fatigue/inertia scores while decreasing water intake of high-consumers (>2 L/day) decreased contentedness, calmness, positive emotions, and vigor/activity scores without affecting sleepiness [49]. Finally, fluid deprivation for 24 h did not affect sleepiness [54]. 

In general, our assessment is consistent with the conclusions from the aforementioned reviews, whereby hypohydration and/or thirst is consistently associated with increased negative emotions. The effect of hypohydration on attention and memory seem to suggest that >1% body mass loss is associated with deterioration in attention and memory, although this may be subdomain- and/or sex-dependent. Fatigue/tiredness appears to be rated higher with dehydration and is unlikely to be affected by acute water consumption. Data on other domains of cognition and sleepiness are sparse and require further research.

#### 3.2.2. Cognition and Mood in Children

Our search identified nine studies, which are summarized in Table 2. Similar to data for adults, results from studies on hydration and cognition and mood in children are mixed. Studies in children have reported improvements in visual attention [38,41,44,45,54], but not sustained attention [46] following acute water consumption. The effect of acute effects of hydration on memory is dependent on the type of memory assessed, whereby some studies reported improvements in immediate memory [46] and others reporting no effects on verbal memory [38], visual memory [44], and story memory [45]. For the aforementioned acute studies, although a majority of studies did not assess baseline hydration status, it is likely that the children were mildly hypohydrated prior to acute water intake. In adolescents (mean age of 16.8 y), dehydration induced by thermal exercise (mean body mass loss of 1.7%) did not affect executive function, although brain imaging demonstrated increased fronto-parietal brain activation during the cognition task, suggesting a need for greater mental effort when dehydrated [43]. 

For longer-term water consumption (i.e., one whole day), results were mixed. Short-term memory assessed by auditory number span improved with additional water consumption (average 624 mL over a school day) in one study [42], but was not replicated in another study using digit recall [39], although exact amount of water consumed was not reported in the latter study. Other cognition domains including visual attention, selective attention, visual memory, visuomotor skills, perceptual speed, and verbal reasoning were unaffected by additional water consumption throughout the day [42,39]. 

Data on mood in relation to hydration status is also limited in children. Mood assessed using the POMS questionnaire did not change following additional water consumption for one day [42] while subjective ratings on happiness were not significantly affected by acute water consumption [45].

Overall, acute consumption of fluid by children appears to improve visual attention, with data on sustained attention being mixed. The effect of acute and chronic fluid consumption on memory is sparse and inconsistent. Finally, the limited data available on hydration and children suggest that hydration does not affect mood.

#### 3.2.3. Headache

Hypohydration is thought to be a cause of headache, and increased fluid consumption has been suggested to relieve some forms of headache. However, evidence on hydration and headache is limited. The two intervention trials that were conducted by the same group were identified, with the earlier report describing a pilot assessment for the latter report, which was a larger trial [65,66]. Results from the two-week pilot study on migraines in adults were promising, with observed reductions in total hours of headache and mean headache intensity in the subjects who drank additional 1.5 L/day water compared to a control group who were given a placebo tablet [66]. In the follow-up study, a larger intervention trial, drinking more water (additional 1.5 L/day) resulted in a statistically significant improvement of 4.5 points on the Migraine-Specific Quality of Life scale [65]. Almost half (47%) of the subjects in the intervention (water) group self-reported improvement against 25% of the subjects in the control group [65]. However, objective measures such as headache days, hours of headache, and medication use were not different between subjects who consumed additional water and controls [65]. The authors noted several limitations in the larger intervention study, including partial unblinding of subjects, small sample size, and a large attrition rate [65].

### 3.3. Renal Function

A common disorder discussed in reviews found on hydration and kidney/renal function is kidney stones, which affects up to 12% of the world population [67]. Observational studies report an association between low total fluid intake and high risk for kidney stones, leading to guidelines recommending increasing fluid intake as a preventative strategy against kidney stones [68,69]. Although our search strategy was not designed to target any specific renal condition, only studies on kidney stones remained after filtering out diseases/disorders that are not relevant to the general population (e.g., chronic kidney disease). We identified one meta-analysis on high fluid intake and kidney stones which reported a significant association between high fluid intake and a lower risk of incident kidney stones, with 0.40-fold (RCTs) and 0.59-fold (observational studies) decreased risk [27]. In addition, high fluid intake reduced the risk of recurrent kidney stones (RR 0.40) [27]. With the exception of providing an explicit statement of questions being addressed and clarifying if a review protocol existed, the meta-analysis fulfilled all required PRISMA reporting items (Table 1). The meta-analysis fulfilled 10 out of the 16 required AMSTAR 2 items, but lacked clarity on study selection and completeness in assessment of included studies.

There have been very few intervention studies measuring the effect of hydration on kidney stones. We identified two relevant studies and these were already included in the aforementioned 2016 meta-analysis. In a 5-year randomized study, patients with idiopathic calcium stone disease had a 12% recurrence rate when encouraged to increase their fluid intake to achieve a urine output of 2 L/day, and a 27% recurrence rate if they were not given specific advice on urine output [70]. Another study investigated the effects of increased fluid intake (to achieve urine output of at least 2.5 L/day) following shock wave lithotripsy (SWL) treatment in stone patients. Among those who were stone free following SWL treatment, rate of recurrence was 8.3% for those with increased fluid intake, compared to 40% for those who were taking Verapamil, a calcium entry blocking agent, and 55% for those who were not provided any specific medication or dietary instructions [71]. Although not statistically significant, the rate of stone regrowth among those with residual fragments following SWL was lowest in subjects with increased fluid intake compared to those who received Verapamil (15.3% vs. 20%, respectively) [71]. Subjects who did not receive any intervention had a regrowth rate of 64% [71].

### 3.4. Gastrointestinal Function

Our search on hydration and gastrointestinal function resulted in one review that addressed the role of fluid intake in the prevention and treatment of functional intestinal constipation in children and adolescents [28] (Table 1). One review was found on the effect of beverage types on gastric emptying and subsequent nutrient absorption [72]; however, this is outside the scope of our review as it did not address hydration alone. Following screening, we found four intervention studies on constipation and one study that assessed the effect of dehydration on gastrointestinal function at rest in humans (Table 3). Our search strategy also resulted in a number of intervention studies that compared different types of beverages on exercise-induced gastrointestinal dysfunction and dehydration, and as noted in the methods, these were not considered within scope of the present review. 

The review on functional intestinal constipation in children and adolescents included 11 studies that either evaluated fluid intake as a risk factor for constipation or evaluated the role of fluid intake in the treatment of intestinal constipation in children or adolescents [28]. Authors reported the possibility of a causal association between lower fluid intake and constipation but noted that study outcomes were heterogeneous and thus, difficult to compare [28]. For the most part, studies that assessed fluid intake as a treatment of constipation showed no effects, although authors again noted the heterogeneity in methodologies of the studies [28]. Of the four intervention studies on constipation, two reported beneficial effects of increased fluid intake on stool measurements and the other two reported no effects (Table 3). The largest trial involved 117 adults with chronic functional constipation randomized to receive 25 g/day fiber alone (with ad libitum fluid intake) or 25 g/day fiber alone with 2 L/day water for 2 months [73]. The water supplemented group consumed more fluid (mean of 2.1 L/day vs. mean of 1.1 L/day) and had greater stool frequency and fewer use of laxatives compared to the ad libitum group [73]. In another study, healthy men were prescribed standardized nutritional and physical activity regimens and randomized to 0.5 L or 2.5 L of fluid per day for one week followed by a crossover after a two-week washout period [74]. During periods of fluid restriction, authors observed reduction in stool weight and frequency and increased tendency towards constipation [74]. When regular fluid consumption was resumed, bowel function returned to normal [74]. In contrast, the two other intervention studies in adults did not report changes in stool measurements following additional fluid intake. Consumption of additional 1 L/day for the first two days followed by 2 L/day for the next two days of either near isotonic fluid or hypotonic fluid (i.e., water) increased urinary output but did not affect stool weight in healthy adults [75]. Addition of 15 g/day wheat bran for 14 days slowed gastric emptying, shortened oroanal transit, and increased stool frequency and stool weight; however, the consumption of 600 mL fluid with the wheat bran did not affect these measurements when compared to consumption of wheat bran alone [76].

Finally, heat-induced dehydration of 3% body mass loss decreased gastric emptying compared to euhydration conditions, but did not affect orocecal transit time, intestinal permeability, and intestinal glucose absorption in healthy men (*n* = 10) [77]. 

### 3.5. Body Weight and Body Composition

Studies on beverage consumption and weight management have mainly focused on the replacement of caloric beverages with non-caloric or lower calorie beverages. A 2016 systematic review on water intake and body weight/weight management was identified [29], which provides a comprehensive listing of the human intervention studies published through 2014 that assessed water intake on energy intake, energy expenditure, body mass index (BMI), and weight change. The review included 134 total RCTs representing 440 different test conditions. Only a handful of these studies investigated the effects of water intake on body weight and body composition independent of changes in caloric intake and physical activity. Two were studies in adults [78,79] and two were in children [80,81]. Akers et al. [78] reported reductions in body fat, but not body weight or BMI, in overweight and obese adults who consumed ~3 times more water compared to a control group (average 1241 g/day vs. 451 g/day, respectively). In this study, energy intake of the water group was slightly greater than that of the control group, although these were not significantly different (average 1726 kcal/day vs. 1654 kcal/day).In another study, adults who were assigned a hypocaloric diet and 500 mL water prior to each daily meal lost more body weight and total fat mass compared to those on a hypocaloric diet alone [79]. Energy intake significantly decreased by the end of the 12-week intervention but was not different between water and control groups (average intake at 12 weeks: 1454 kcal/day vs. 1511 kcal/day, respectively) [79]. Additionally, ad libitum meal intake assessed at the end of the intervention was not different between groups, with or without 500 mL water pre-load [79]. In an 8-week intervention study, children (BMI percentile of ≥ 85%) who replaced caloric beverages with water and increased water consumption lost more body weight compared to children who only replaced caloric beverages with water [81]. Of note, at the end of the study, urine osmolality was below 500 mmol/kg in the group that increased water consumption, while urine osmolality stayed above 500 mmol/kg in the group that only replaced caloric beverages with water [81]. Increased water consumption (+1 glass/day) following a water intake promotion program for 1 year did not result in changes in BMI-z scores in students, although the percentage of students who were overweight was lower in the intervention group compared to the control group [80].

Our updated search resulted in 549 titles (Figure 1), and the majority of these were excluded because they investigated the effect of replacement of caloric beverages with non-caloric beverages, replacement of non-caloric beverages with water, or methodological considerations of hydration on BMI assessments. When compared with the 2016 systematic review [29], our search found four new publications; of which one [82] was a duplicate publication of a study that was already included in a previous systematic review [83]. The three new studies (Table 4) varied in design, with one being a study on hydration status and energy intake [84], one on water preloading and weight loss [85], and the other on increased water consumption and weight loss [86]. Of the new studies, one investigated the very short-term effect (i.e., 24 h) of euhydration vs. hypohydration on ad libitum breakfast energy intake in healthy men and observed no difference in energy intake between groups [84]. Another reported greater weight loss following water pre-loading (500 mL) before main meals for 12 weeks in obese adults (*n* = 84, [85]). Finally, increasing water consumption (mean increase of ~310 mL/day) did not affect BMI and other anthropometric measures in overweight and obese adolescents (*n* = 38) who were enrolled in a weight-loss program for 6 months [86]. The results of these new studies were mostly consistent with the general observations presented by the 2016 systematic review. The long-term study that reported weight loss instructed obese subjects to follow an energy-restricted diet and consume >1 L water/day, although the change in glucose and insulin is unknown [85]. In contrast, the authors of the long-term study that did not observe changes in body weight commented that subjects failed to increase water consumption, such that there were no differences in urine specific gravity between the water and control groups [86].

## 4. Discussion

Water is involved in virtually all bodily function. Thus, ensuring that the body has enough water to maintain proper function is important for health. According to the analysis of combined urine osmolality data from the NHANES 2009–2010 and 2011–2012 surveys, about 1/3 (32.6%) of adults (ages 18–64 years old) [87] and more than half (54.5%) of children and adolescents (ages 6–19 years old) [88] in the US are inadequately hydrated. Therefore, it is important to understand the effect of hydration on health in the general population. This review is a compilation of evidence on hydration and various health outcomes thought to have a beneficial effect among the general population, including skin health, gastrointestinal and renal function, cognition, mood, headache, and body weight and composition, with cognition being the most researched. Overall, there is a growing body of evidence supporting the importance of maintaining a normal state of hydration on various health aspects, although the strength and quality of the evidence vary within each health area (Table 5). 

Evidence on hydration status and skin health is limited and no new studies published after a 2018 systematic review of the topic [24] were identified. Results from the handful of studies included in the review suggest that increasing water consumption may improve SC hydration. One of the most important functions of the skin is its ability to serve as an efficient barrier to molecular diffusion and the SC layer of the epidermis is the primary location of this barrier function. SC hydration is intimately related to the structure and function of the SC [89], thus, it is often an outcome in studies on skin health. The improvements in SC hydration following increased water consumption reported by existing intervention studies suggest better skin barrier function with increased oral hydration; however, these studies reported no changes in TEWL, which is another measure of skin barrier integrity. Therefore, the effectiveness of additional water consumption on skin barrier function is unclear. Furthermore, these studies failed to consistently assess other skin parameters such as those related to elasticity, firmness, roughness, surface texture, and pigmentation. Also, the applicability of the results of these studies is unclear; in most cases, subjects were already meeting the water intake recommendations and/or were required to consume water above the recommended intakes. Available studies were assessed to have low methodological quality and extremely high risk of bias by the authors of the systematic review [24]. 

Studies on hydration and neurological function focused on cognition, mood, and headache, with some also assessing sleep and fatigue. Studies on cognition investigated a variety of subdomains using different assessment tools, which makes comparisons across studies challenging. Evidence within each subdomain is sparse; thus, the specific influence of hydration on cognition is unclear. Reviewing the evidence in adults and children together, however, suggests that hypohydration negatively influences attention and results in the need for greater effort when performing attention-oriented tasks, which is ameliorated by rehydration. The effects of hypohydration and rehydration are less pronounced for memory and reaction time. Our observations are consistent with results from a recent meta-analysis published after our search date of April 2018 [90]. The meta-analysis authors reported that high-order cognitive processing (involving attention and executive function) and motor coordination appear more susceptible to impairment following dehydration compared to other domains involving lower order mental processing (e.g., simple reaction time) [90]. Additionally, across all cognitive domains and outcomes, studies eliciting a >2% body mass loss resulted in significantly greater cognitive impairments than studies eliciting ≤ 2% body mass loss [90]. The relationship between hydration and mood appears to be more consistent in adults, with hypohydration associated with increased negative emotions such as anger, hostility, confusion, depression, and tension as well as fatigue and tiredness. In children, however, data on hydration and mood is very limited and unclear. Overall, hydration does affect cognition and mood, although the specifics of the relationships are unclear. Finally, the evidence is too limited to determine if hydration affects headache. Two studies reported that increases in water consumption did not improve objective measures of headache, including number of days with headaches, hours of headache, and medication use, although subjective measures, such as headache intensity rating and quality of life questionnaire scores, were improved.

The renal system plays an important role in maintaining water and salt homeostasis; thus hydration is often associated with renal function and health, particularly the risk of kidney stones. The pathophysiology of kidney stone development is not yet fully understood, although a likely cause is an acquired or congenital anatomical structural abnormality [91]. Kidney stones form when the concentration of stone-forming salts exceeds their saturation point in the urine [91]. Thus, it is commonly suggested that dehydration may lead to the development of kidney stones, and that consumption of fluid may decrease the risk of kidney stones. Findings consistently suggest that increased fluid intake resulting in increased urine output is related to reduced risk of kidney stone development or recurrence rate, although data are limited. For example, a recent meta-analysis that included two intervention and seven observational studies reported a significant association between high fluid intake and a lower risk of incident kidney stones [27]. The observational studies were of moderately high quality, however, the two intervention studies were of low quality [27]. Further, limitations in the current literature include differences in diagnostic methodology of kidney stones, as well as inconsistent definitions of stone occurrence and recurrence. Overall, kidney stone occurrence is likely dependent on hydration and additional intervention studies are needed to confirm this relationship.

Another important kidney function is the removal of a wide variety of potentially toxic xenobiotics, xenobiotic metabolites, as well as metabolic wastes from the body. Elimination of unwanted substances via the urine depends on several variables that are, in turn, highly dependent on hydration status. These include renal blood flow, the glomerular filtration rate, the capacity of the kidney to reabsorb or to secrete drug molecules across the tubular epithelium, urine flow, and urine pH [92]. Because of this, detoxification is commonly associated with fluid intake or hydration. In conventional medicine, toxins generally refer to drugs and alcohol, and ‘detox’ is the process of weaning patients off these addictive substances. Commercially (and among laypersons), “detox” often refers to the removal of substances that may include, but are not limited to pollutants, synthetic chemicals, heavy metals, and other potentially harmful products of modern life. Little, if any, evidence exists to support the use of commercial detox diets for toxin elimination [93].

Increased fiber and fluid consumption are typical dietary-based therapeutic approaches to functional constipation [94,95]. Results from epidemiological studies suggest a possible relationship between low fluid intake and intestinal constipation occurrence. However, clinical trials currently do not support the use of increased fluid intake in the treatment of functional constipation. A comprehensive review of hydration and constipation in children and adolescents reported that fluid intake was ineffective in treating constipation [28]. Of the intervention studies we identified, one was in adults with chronic constipation and the rest were in healthy adults. Increased water intake by adults with chronic constipation increased stool frequency and decreased laxative use, while additional water consumption by healthy adults did not affect stool outcomes, suggesting a possible effect of increased hydration and improvements in stool output only in those with existing constipation. Further studies are necessary to better understand the role of water and fluids in the etiology and treatment of intestinal constipation, and study designs should include standardized evaluation tools for constipation outcomes (e.g., stool consistency, frequency of bowel movement), control for confounding dietary and lifestyle factors (e.g., fiber intake, physical activity), and measurements of hydration status and fluid intake. Additionally, published studies investigating the effect of hydration on normal functions of the gastrointestinal tract in healthy humans are lacking. Studies on dehydration and gastrointestinal function have mainly focused on exercise or gastrointestinal disorders [77].

A majority of the studies on fluid intake and weight management focused on the replacement of caloric beverages with non-caloric beverages and this has consistently resulted in lower overall energy intake [29]. However, consumption of hypoosmotic solutions such as water may contribute to weight loss by increasing lipolysis, fat oxidation, and thermogenesis, independent of changes in caloric intake as suggested by animal studies [96]. Only a handful of studies have investigated the influence of fluid intake (specifically water intake) on changes in body weight and/or body composition, independent of changes in energy intake. Existing data in adults suggest that increased water intake contributes to reductions in body fat and/or weight loss in obese adults, with or without a hypocaloric diet. Data in children/adolescents are less clear, possibly due to the smaller difference in water consumption between control and intervention groups in these studies (~350 mL/day) compared to the studies in adults (>800 mL/day). Adherence to the hydration regimen is a common problem reported by intervention studies in children/adolescents. Additionally, background diets were not collected in these studies, with the authors citing limitations in collecting dietary records in children/adolescent as the reason. While existing evidence show promising results for hydration and weight management, more studies are needed to confirm and clarify the effect of water intake on body weight and composition in adults and children.

Overall, assessing the totality of the hydration evidence is challenging due to the diversity in population, interventions, and trial designs across the studies. While many studies were conducted using a dehydration-rehydration design, variations in dehydration protocols (e.g., passive or active dehydration, type of exercise and environment, length of passive dehydration, extent of dehydration) and rehydration strategies (e.g., amount and timing of fluid intake) were observed. Outcome assessment tools, particularly for cognition, varied greatly, making it difficult to obtain enough evidence to clearly determine the impact of hydration on specific cognition subdomains. One of the biggest challenges with hydration studies is achieving consistency in the way hydration, dehydration, and overhydration is defined and measured. This is further complicated by the current lack of widely accepted screening tools or gold standard tests that allow for easily performable and replicable measurements of fluid balance and fluid intake. Another challenge for hydration studies is the difficulty in blinding subjects to the intervention. Possible solutions include providing intravenous fluid instead of oral hydration, although this bypasses the body’s normal indicators of hydration status such as thirst and oropharyngeal reflexes, which may play a role in the effect of hydration on various outcomes. Therefore, it is even more important that future studies ensure blinded assessment of study outcomes.

An important gap in knowledge is the effect of small variations in hydration on health in the general population. The point at which dehydration (or rehydration) affects health indicators is not easily determined from the current body of literature. Understanding the effect of different levels of mild dehydration on health is important as a substantial number of the general US population, especially older adults, drink less than the Adequate Intake for water that was established by the IOM [87,88,97]. Another gap in knowledge is the influence of sex on the effects of hydration on health as only a minority of the studies found considered both males and females. Additionally, there is also a need to consider the stage of the menstrual cycle as female sex hormones (estrogen and progesterone) are known to influence body fluid regulation [98,99]. These are particularly important considerations for studies in which outcomes are also known to be influenced by hormones, such as cognition and mood. Finally, understanding how hydration affects health in older adults and children is also important. Many of the health outcomes discussed in this review such as weight loss, cognition, kidney stones, and constipation are highly relevant to older adults and children. Older adults are susceptible to dehydration [100] due to various physiological (blunted thirst response, decline in kidney function) and environmental (limited mobility, inadequate assistance in nursing homes or hospital stays) factors [14], which can then lead to increased morbidity [101,102]. Meanwhile, as reviewed here, dehydration in children may have a negative impact on cognitive development and school performance in addition to physical health. 

The goal of this narrative review was to present the state of the science on hydration that is relevant to the general population. Although a systematic approach was used to identify the literature and the search was broad, the publications included may not represent all available studies and reviews on the effects of hydration and specific health areas, given only one database was used, non-English publications were excluded, and the possibility that the search terms did not reflect all relevant conditions. Finally, the studies were quite heterogeneous, making broad conclusions difficult.

## 5. Conclusions

Water is the largest single constituent of the human body, making up approximately 60% and 75% of the adult and child human bodies, respectively. Body water deficits challenge the ability to maintain homeostasis during perturbations (e.g., sickness, physical exercise, and environmental exposure) and can affect function and health. As shown in this review, hydration status is an important aspect for health maintenance; however, evidence on the specific effects of hydration relevant to the generally healthy population is scarce and mostly inconsistent. The relationships between hydration and cognition, kidney stone risk, and weight management in generally healthy individuals are perhaps the most promising, although additional research is needed to confirm and clarify existing findings. Additional high-quality studies are needed to fill current gaps in knowledge and enable us to understand the specifics on the role of hydration in promoting health, as well as to help inform public health recommendations. 

## Figures and Tables

**Table 1:** Summary of key hydration reviews.

Citation	Review Type/ Reporting Quality	Description	Key Findings/ Conclusions
**Skin Health**			
Akdeniz et al., 2018 [24]	Systematic review Met 17 of 19 required PRISMA items for systematic review. ^1^ Fulfilled 4 of 13 required AMSTAR 2 items for systematic review.^2^	Assessed fluid intake and skin hydration and/or barrier function. Included human intervention studies published through 2016. Met PRISMA reporting criteria except for absence of explicit statement of questions being addressed. Risk of bias for each study assessed using Cochrane Risk of Bias tool. 5 intervention studies included.	Additional dietary water intake is associated with increased skin hydration and reduced skin dryness. Evidence is weak overall in terms of quantity and methodological quality and the clinical relevance is unclear.
**Neurological Function**			
Benton and Young, 2015 [25]	Narrative review	Assessed relationship of hydration and mood and/or cognition. Information on search strategy, selection process, and data extraction were not provided. 30 intervention studies included.	Data consistently show a reduction of >2% in body mass due to dehydration results in effect on mood, fatigue, and alertness. Effects on cognition have been less consistent. Only a few studies have looked at females, and due to the effect of sex hormones on kidney function, this is a gap in knowledge. Four intervention trials on cognition in children show an effect of hydration. Lack of studies in sedentary adults living in temperate climates, as well as older adults.
Masento et al., 2014 [26]	Narrative review	Assessed relationship of hydration and cognition. Search strategy not provided in full detail and no information on selection process and data extraction were provided. 22 intervention and 4 observational (prospective cohort and cross-sectional) studies included.	Data suggests hydration is important for supporting cognition and mood. Hydration is particularly important in those with poor fluid regulation, which includes children and elderly. Challenges in quantifying effects across studies include a lack of standardized approaches to assessing cognition, as well as difficulties in assessing hydration state.
**Renal Function**			
Cheungpasitporn et al., 2016 [27]	Systematic review/Meta-analysis Met 22 of 24 required PRISMA items for meta-analysis.^1^ Met 10 of 16 required AMSTAR 2 items for meta-analysis. ^2^	Assessed fluid intake and kidney stones, as well as adherence and safety of high fluid intake to prevent kidney stones. Included RCTs and observational studies published from 1980 through 2014. Met PRISMA reporting criteria except for absence of explicit statement of questions being addressed. Risk of bias for each study assessed using Jadad score. 2 RCT, 6 prospective cohort, and 1 cross-sectional studies included.	Significant association between high fluid intake and a lower risk of incident kidney stones based on pooled risk ratio (RR) for RCT only [0.40 (95% CI 0.20–0.79)] and observational studies only [0.49 (95% CI 0.34–0.71)]. A subgroup analysis found that high fluid intake was associated with decreased kidney stone risk in both men [RR 0.67 (95% CI 0.58–0.79)] and women [RR 0.65 (95% CI 0.56–0.76)]. RCTs were graded as low-quality and observational studies were graded as moderately high, with detectable heterogeneity among observational studies.
**Gastrointestinal Function**			
Boilesen et al., 2017 [28]	Narrative review	Assessed the role of water and fluid intake in the prevention or treatment of functional intestinal constipation in children and adolescents. Information on search strategy, selection process, and data extraction provided, albeit not in full detail. Included epidemiological and clinical studies published from 1966 through 2016. 5 intervention, 5 cross-sectional, and 1 case-control studies included.	Studies with a sample of the general population suggest that a lower intake of water and/or fluids is associated with intestinal constipation; however, those with existing constipation did not show an advantage of greater fluid intake.
**Body Weight and Body Composition**			
Stookey, 2016 [29]	Qualitative review	Assessed totality of evidence on hydration and weight-related outcomes. Included human intervention studies published through 2014. Search strategy not provided in full detail but selection process and data extraction were fully explained. 134 RCT included on hydration and energy intake, energy expenditure, fat oxidation, and weight change.	Drinking water increases energy expenditure in metabolically-inflexible, obese individuals. Drinking water increases fat oxidation when blood sugar and/or insulin are not elevated and when it is consumed instead of caloric beverages.

Abbreviations: AMSTART, A MeaSurement Tool to Assess systematic Reviews; CI, confidence interval; PRISMA, Preferred Reporting Items for Systematic Reviews; RCT, randomized controlled trial; RR, relative risk. ^1^ PRISMA is an evidence-based minimum set of items for reporting in systematic reviews and meta-analyses and has been used to assess reporting quality. For meta-analysis, the PRISMA checklist contained 24 required reporting items that were used to assess quality. For systematic reviews, 19 items remained after exclusion of items specific to meta-analyses (i.e., item 13, 14, 15, 21, and 22 which are related to data analysis and overall risk bias assessment). ^2^ AMSTAR 2 is an instrument used to assess the methodological quality of systematic reviews and meta-analysis. It has 16 items in total, whereby three of these are specific for meta-analysis. AMSTAR 2 is not intended to generate an overall score and thus, none is provided.

**Table 2:** Intervention Studies on Hydration and Neurological Function ^1^.

Citation	Objective	Population	Design	Intervention/Control	Summary/Conclusion
**Neurological Function in Children**					
Edmonds et al., 2017 (Children) [38]	Examine dose–response effects of water on cognition in children	60 children (58% F) ages 7–10 years.	Acute RCT	Children consumed the assigned water 20 min prior to cognition tasks: 0 mL water (*n* = 20, 10/10 M/F) 25 mL water (*n* = 20, 10/10 M/F) 300 mL water (*n* = 20, 5/15 M/F)	No significant time × volume interaction for visual attention (Letter cancellation task) and working memory (digit span task). Significant increase in pre- and post-water scores for visual attention for 25 mL and 300 mL with *t*-test analysis. No significant results on memory task.
Trinies et al., 2016 [39]	Assess the role of hydration on cognition in children living in hot, low income environments	279 children in grades 3–6 in schools across Eastern Zambia.	Parallel RCT	Students in area where water was not nearby were provided: No water bottles (*n* = 143, 68/65 M/F; Control group) Refillable water bottle (water group, *n* = 149, 61/82 M/F) Ambient temperature was 26.6–35 °C, and humidity ranged from 7–28%. Cognition was tested in the morning and after lunch on the day of intervention.	Afternoon hypohydration, assessed by mean urine specific gravity, was lower in children provided water (9.8%) compared to the control children (67.2%). No significant difference between groups in visual memory (indirect image difference), short-term memory (forward digit recall, and reverse digit recall), or visuomotor skills (line trace). Two visual attention tests were included, with a significant difference in one visual attention test (direct image difference; *p* = 0.05), but not the other (letter cancellation). When grouped by urine specific gravity (≥1.020 as cut-off for hypohydration) no significant difference was observed.
Perry et al., 2015 [40]	Assessed whether the benefit of drinking water on working memory and attention depends upon children’s hydration status and renal response.	52 children (50% F) age 9–12 y.	Acute crossover	All subjects performed a baseline cognition test after standard breakfast (included 25 mL water and 250 mL skim milk). During the water intervention, subjects consumed 250 mL water, followed by cognition test battery (short-term effect), and another 500 mL water over the next 3 h, followed by another cognition test batter (long-term effect). During the control intervention, no additional fluid beyond breakfast was provided.	Based on osmolality, 65% of the population was dehydrated prior to breakfast intake, with 35% remaining dehydrated at the end of the control test period compared to 3.8% at the end of the active (water) period. Children who exhibited smaller decreases in urine osmolality following water intake (i.e., had underlying hypohydration) performed significantly better on the water day compared to the control day on the digit-span task (verbal memory) and pair-cancellation task (sustained attention). Children who exhibited larger decreases in urine osmolality following water intake performed better on the control day compared to the water day. No significant effects on working memory (tested using delayed match-to-sample task).
Booth et al., 2012 [41]	Investigate the effects of water supplementation on visual attention and motor performance in schoolchildren.	15 students (age 8–9 y)	Acute RCT	Children were provided no water or 250 mL bottle of water and instructed to drink as much or as little 20 min prior to cognition test and mood ratings.	When offered water, children drank an average of 168 mL water. Children performed better on tasks testing visual attention and fine motor skills (Letter Cancellation Task and Wii Ravin Rabbids game) after water consumption compared to no water consumption. There were no differences for tasks testing gross motor skills (Ball Catching and Step Ups) and happiness rating.
Fadda et al., 2012 [42]	Assessed the effect of drinking water on cognitive performance, fatigue, and vigor in school children.	168 children age 9–11 y living in a hot climate (Southern Italy).	Parallel RCT	Control group (*n* = 75, 35/40 M/F) and a supplemental water group (*n* = 93, 47/46 M/F). The water group received 1 L of additional water for the day; the control group did not receive additional water.	Based on urine osmolality measurement, 84% of children were dehydrated (morning Uosm >800) at the start of the school day. Drinking water benefited short-term memory (auditory number span) and verbal reasoning (verbal analogies) but not selective attention (Deux de Barrage). No significant differences were found in POMS fatigue or confusion scores. Significant beneficial relationship between hydration and vigor was noted.
Kempton et al., 2011 [43]	Investigate the effects of dehydration on brain function	10 healthy adolescents (50% females), average age 16.8 y	Acute RCT	Subjects consumed 500 mL of water the evening prior to test day. On test day, subjects consumed a further 500 mL of water 1 h before a 90-min thermal exercise dehydration protocol (with thick and multilayered clothing) or a 90-min non-thermal control exercise protocol.	Subjects lost an average 1.65% body mass during the thermal dehydration exercise compared to 0.53% during the non-thermal control exercise. Hypohydration resulted in increased fronto-parietal brain activations during a task of executive function, lateral ventricular volume, and mental and physical sedation, but did not affect results of the executive function task.
Edmonds and Jeffes, 2009 [44]	Assess the effect of water consumption on cognition in children.	23 children (61% female) age 6–7 y from one classroom.	Acute parallel	Children were separated into water group (*n* = 11, 4/7 M/F) or no water (*n* = 12, 5/7 M/F). Children were tested for baseline function as a group, then the no water group left the room and those remaining were provided 500 mL water to drink ad libitum. The post-consumption test occurred 45 min later.	Significant positive changes were reported for children consuming water on the thirst and happiness ratings, as well as the visual attention and visual search tests. Visual memory and motor performances were not significantly different. Although there was an effect on mood, the authors caution making strong conclusions based on this finding due to the lack of significance on follow-up testing.
Edmonds and Burford, 2009 [45]	Assessed the effect of a drink of water on children’s cognitive function.	58 children, age 7–9 y.	Acute RCT	Control (no water) group (*n* = 30, 15/15 M/F) and a water group (*n* = 28, 11/17 M/F). Children were tested at baseline and 20 min later. Children in the water group were provided with 250 mL of water between tests.	Children who drank additional water rated themselves as significantly less thirsty and performed better on letter cancellation task (visual attention) and spot the difference task (visual attention and memory). There were no differences between groups for story memory and visuomotor tracking tasks.
Benton and Burgess, 2009 [46]	Examined the influence of giving additional water to school children on measures of memory and attention.	40 children (45% female) in a school in South Wales, average age 8 y.	Acute RCT	Children were tested in the afternoon after receiving 300 mL of water or no water at the beginning of the mid-afternoon break, with testing occurring 20–35 min after consumption.	Immediate memory (recall of objects) was significantly better from children after consumption of water. The ability to sustain attention (paradigm of Shakow) was not significantly influenced by water consumption.
**Neurological Function in Adults**					
Stachenfeld et al., 2018 [47]	Investigate whether mild dehydration would adversely impact executive function tasks, with no effects on simple tasks, and that these changes in cognitive performance are independent of changes in emotion	12 healthy women (age 18–34 y)	Crossover RCT	Subjects performed cognitive tasks and rated mood under three difference hydration conditions: Control: Typical water intake (mean of 2.3 L) Dehydration: Water deprivation (mean of 0.7 L) Euhydration: Water intake conformed to EFSA and IOM recommendation (mean of 2.4 L) Control condition was always performed first, and order of dehydration and euhydration was randomized. Tests were performed during early follicular phase of the menstrual cycle, but a week apart for those on hormonal contraception.	Water deprivation increased plasma osmolality from ~283 to 287 Uosm/kg H_2_O. Water deprivation increased errors for tests for visual memory or working memory (Continuous Paired Associate Learning) and executive function and spatial problem solving (Groton Maze Learning Test) when compared to control and euhydration conditions. No hydration effect on simple reaction time, choice reaction time, visual attention, motor speed, visual motor function, visual learning, working memory assessed with One and Two Back Tasks, and cognitive flexibility. There were no changes in mood outcomes.
Edmonds et al., 2017 (Adults) [38]	Evaluate the dose–response effect of water on cognitive performance and mood in adults	96 adults, average age 21 y.	Acute RCT	Acute consumption of 300 mL water (*n* = 32, 10/22 M/F), 25 mL water (*n* = 32, 7/25 M/F), and 0 mL water (*n* = 32, 11/21 M/F) 20 min before cognition tasks.	Significant time × volume interaction for visual attention (letter cancellation task), whereby scores increased from baseline in a dose-dependent manner, with 0 mL having the lowest increase and 300 mL having the highest increase. Significant time × volume interaction for working memory (digit span task). Only the increase in the 300 mL group was significant for the memory test.
Benton et al., 2016 [48]	Assess whether a loss of 1% of body mass due to hypohydration adversely influenced cognition, and examined the possible underlying mechanisms	101 healthy adults. Water group aged 18–30 y; control group age 18–31 y.	Acute RCT	Water consumption group (*n* = 51, 26/25 M/F) and no water consumption group (*n* = 50, 26/24 M/F). Subjects were exposed to 30 °C for 4 h, during which they either did or did not drink 300 mL pure water.	Subjects in the no water group had greater body mass loss (−0.22% vs. +0.05%) and increase in osmolality (−117.24, no water vs. water). At 90 and 180 min, water consumption resulted in better episodic memory (word list recall task) and focused attention (arrow flanker test). Energy and depression ratings were unaffected by water consumption. Anxiety rating decreased with water consumption at 90 min, but not 180 min.
Pross et al., 2014 [49]	Evaluate effects of changing water intake on mood and sensation in habitual high- and low-water consumers.	52 subjects (79% F) average age 25 y were selected based on daily fluid consumption: Low <1.2 L/d (average 1.0 L/d) High ≥2 L/d (average 2.5 L/d)	Open label 2-d intervention	Intervention conducted in controlled setting (inpatient facility) with meals (details not provided) and sleep/wake cycles standardized. Baseline data were collected during days 1–2, and intervention conducted days 3–5. Defined drinking programs were: Low consumers increased water intake to 2.5 L/d (11/19 M/F) High consumers reduced water intake to 1L/d (0/22 M/F)	Increasing water intake decreased urine osmolality from mean of 841 to 392 mOsm/kg. Decreasing water intake increased urine osmolality from mean of 222 to 720 mOsm/kg. At baseline, POMS ratings were comparable except for thirst and some depression scores. Restricting water intake in high-consumers resulted in a significant increase in thirst and decrease in contentedness, calmness, positive emotions, and vigor/activity scores. Increasing water intake in low consumers significantly decreased fatigue/inertia, confusion/bewilderment, and thirst scores, with a non-significant decrease in sleepiness.
Edmonds et al., 2013 [50]	Explore the effects of water and knowledge of aims of study on cognitive performance	44 adults age 18–57 y.	Acute RCT	No water, no expectancy condition (*n* = 9, 3/6 M/F) No water, expectancy condition (*n* = 11, 6/5 M/F) Water, no expectancy condition (*n* = 14, 5/9 M/F) Water and expectancy condition (*n* = 10, 1/9 M/F) Subjects in the water groups were provided 200 mL water prior to mood and cognitive testing. Subjects in the expectancy groups were told that water consumption is believed to aid cognitive performance. Subjects in the no water groups were not provided water. Subjects in the no expectancy groups were not informed of the connection between water and test outcomes.	There were no differences in the amount of water consumed (out of 200 mL) between the water + expectancy vs. the water + no expectancy groups. Visual attention (letter cancellation task) improved with water consumption, regardless of expectancy condition. Working memory (backwards digit span task) was better in the no water groups vs. water groups. There was no effect of expectancy condition. Water and expectancy condition did not affect scores for simple reaction time or mood assessed using VAS. Water consumption effects on visual attention are due to the physiological effects of water, rather than expectancies about the effects of drinking water.
Lindseth et al., 2013 [51]	Examine the effect of fluid intake and possible dehydration on cognitive flight performance of pilots	40 healthy pilots (average age 20.3 y) enrolled during the third term of the commercial phase of their collegiate aviation program at a Midwestern university	Crossover RCT	High- or low-fluid controlled diets (≥80 oz/d vs. ≤40 oz/d or ≥2.4 L/d vs. ≤1.2 L/d) for 2 weeks, with 2 week washout.	No difference between high and low fluid diets for flight performance (General Aviation Trainer full-motion flight simulator), spatial cognition (Vandenberg Mental Rotation Test), and memory (Sternberg Item Recognition Test). Scores for flight performance and spatial cognition were poorer for pilots who were dehydrated (1–3% body mass loss). Hypohydration did not affect memory.
Edmonds et al., 2013 [52]	Investigate the effect of water supplementation on cognitive performance and mood in adults, and whether subjective thirst moderates the relation between water supplementation and cognitive performance and mood	34 healthy adults (74% F), age 20–53 y.	Acute RCT	Water group consumed 0.5–1 L water prior to cognitive and mood testing. The no water group was not provided water prior to testing.	Water supplementation had a positive effect on simple reaction time, whereby those who were thirsty and did not have water performed more poorly compared to those who were not thirsty. However, the poorer performance of thirsty subjects was “normalized” when they were provided water. No significant results for visual memory, visual learning, immediate and delayed memory, comprehension, learning, acquisition and reversal, sustained attention, forced choice recognition, and choice reaction time. Participants rated themselves as more tired and tense if they were thirsty, and consumption of water did not affect mood ratings.
Ely et al., 2013 [53]	Determine the impact of acute exposure to a range of ambient temperatures (10–40 °C) in euhydration and hypohydration states on cognition, mood and dynamic balance	32 men (average age 22 y)	Acute RCT	4 groups (*n* = 8/group) matched for aerobic fitness. Each group went through euhydration and hypohydration conditions in a crossover fashion, separated by 1 week. All subjects went through a dehydration exercise regimen. For the euhydration condition, subjects were provided water to restore body weight to their pre-dehydration body weight. For the hypohydration condition, water was only provided to ensure that body mass loss did not exceed −4.5%. Following the exercise + water consumption (if needed), subjects rested in preassigned temperatures and performed cognition tasks.	Sustained attention, choice reaction time, short-term spatial memory, and grammar-based logical reasoning were unaffected by hypohydration (4.0–4.2%body mass loss) or ambient temperature during cognition testing. Hypohydration (4.0–4.2%body mass loss) led to increased total mood disturbance, with increased ratings of anger/hostility, confusion/bewilderment, depression/dejection, and fatigue, without affecting vigor/activity and tension/anxiety. Temperature did not affect mood.
Pross et al., 2013 [54]	Assess no drink allowed for 23–24 h	20 healthy women, average age 20 y	Crossover RCT	Subjects completed the following: 24-h fluid deprivation: no beverages from 6–7 p.m. until 6 p.m. next day. Fully hydrated control: water intake at fixed periods. Standardized meals containing (50 g/d water) were provided. 10–30 d washout.	Urine specific gravity significantly increased and color significantly darkened at 9 h and remained so throughout 24 h, but plasma osmolality was unchanged. Generally higher sleepiness and lower alertness scores throughout, but significant at 14–16 h with no fluids. Significantly greater fatigue and lower vigor ratings with no fluids. No significant differences on sleep parameters.
Armstrong et al., 2012 [55]	Investigate if mild dehydration would primarily affect mood and symptoms of dehydration and have modest effects on cognitive function.	25 women, average age 23 y.	Acute RCT	Arm 1: Exercise-induced dehydration + placebo capsule containing no diuretic Arm 2: Exercise-induced dehydration plus a diuretic capsule Arm 3: Euhydration plus placebo capsule. 28 d washout between arms. During the euhydration arm, subjects consumed water equivalent to their body weight loss during and after the exercise bouts.	While in the dehydration arms, subjects lost ≥1% body mass (mean loss of 1.36%) Overall, sustained attention, choice reaction time, working memory, short-term memory, and logical reasoning were unaffected by dehydration. Subjects reported increased anger-hostility, increased fatigue-inertia, and decreased vigor-activity when dehydrated. Total mood disturbance score was worse with dehydration. Subjects also reported greater perceived task difficulty, lower concentration, and increased headache with dehydration.
Ganio et al., 2011 [56]	Assess the effects of mild dehydration on cognitive performance and mood of young males	26 men, average age 20 y	Acute RCT	Arm 1: Exercise-induced dehydration plus a diuretic Arm 2: Exercise-induced dehydration plus placebo containing no diuretic Arm 3: exercise while maintaining euhydration plus placebo. Washout was 4 d.	While in the dehydration arms, subjects lost ≥1% body mass (mean loss 1.59% body mass). Dehydration resulted in lower scores for attention (scanning visual vigilance task) and working memory (matching to sample task) and increased tension/anxiety and fatigue/inertia. No significant results for visual reaction time, choice reaction time, short-term memory, and logical reasoning.
Kempton et al., 2009 [57]	Investigate whether acute dehydration would lead to a reduction in brain volume and subtle regional changes in brain morphology such as ventricular expansion	7 healthy men (mean age 23.8 y)	Single arm	Subjects went through a thermal-exercise dehydration protocol to decrease body mass by 2–3%. Subjects received brain MRI scan before and after the dehydration protocol.	Average body mass loss due to dehydration protocol was 2%. Dehydration led to expansion of the ventricular system with the largest change occurring in the left lateral ventricle, without changes in total brain volume.
Petri et al., 2006 [58]	Measure the deterioration in mental and physical performance and dynamics of its onset during voluntary 24-h fluid intake deprivation.	10 healthy men, age 21–30 y.	Open label 24 h	Testing occurred over 2 d, every 3 h throughout the days for a total of 7 time points a day. During the first 24 h, subjects were allowed to drink freely. From 25–48 h, subjects were not allowed to drink. Daily water intake in food was 500 g. The environmental conditions and diet were controlled.	Speed, accuracy, and mental endurance decreased after 3 h of fluid deprivation. Stability decreased after 9 h of fluid deprivation Energy decreased after 15 h of fluid deprivation. No other effect on mood.
Szinnai et al., 2005 [59]	Determine the effect of slowly progressive dehydration on mental performance	8 healthy women (age 21–34 y) and 8 healthy men (age 20–34 y)	Crossover RCT	During the dehydration arm, subjects abstained from fluid intake for 28 h. During the control arm, subjects were allowed fluid ad libitum.	Urine osmolality increased during dehydration (2.45% body mass loss). Stroop test word naming (verbal response time) revealed significant dehydration-gender interactions, with slower response time in females, but faster response time in males with dehydration. No significant effect of dehydration or sex on visual attention, cognitive-motor speed, sustained attention, and divided attention). No difference with auditory event-related potentials P300. Subjective rating was greater for tiredness and lower for alertness with dehydration.
Shirreffs et al., 2004 [60]	Investigate the physiological responses and subjective feelings resulting from fluid restriction over 37 h compared to euhydration	15 subjects (40% F) average age 30 y	Crossover RCT	Subjects randomized to different hydration conditions for up to 37 h: Fluid Restriction: restrain from all fluid intake and ingest only foods that have low water content. Euhydration: consume normal diet and beverage intake.	Fluid restriction: water from food, 487 ± 335 mL; urinary loss, 1.37 ± 0.39 L; body mass decrease, 2.7 ± 0.6% at 37 h. Subjects reported decreased ability to concentrate, and decreased alertness, and more headaches. Euhydration: water intake, 3168 ± 1167 mL; urinary loss, 2.76 ± 1.11 L.
Neave et al., 2001 [61]	Assess dehydration within normal physiological levels on mood and cognition.	24 generally healthy adults (50% F) average age 20.1 y	Crossover	Subjects did not eat or drink anything from midnight until testing the next morning. Compared 300 mL water vs. no intake (9–11 h no water intake).	Fasting was ~9 h with testing over 2 h. Sustained attention and working memory were not affected by water intake. No water intake negatively affected calmness and alertness, measured using Bond–Lader.
Rogers et al., 2001 [62]	Assessing no drinking vs. 120 mL or 330 mL of water intake	60 adults (50% F), average age 26 y	Parallel RCT	Subjects performed cognition task after acute consumption of the following: Nothing 120 mL water 330 mL water Background diets were customary with testing at 11 a.m. or 3 p.m.	Improved attention with acute fluid intake Increased alertness at 2 min, but not after 25 min. No effect on ratings for revitalization.
Gopinathan et al., 1988 [63]	Determine the effects of various degrees of dehydration on mental performance	11 healthy soldiers (age 20–25 y)	Crossover RCT	Subjects performed moderate work for 2 h under hot and humid conditions (30% relative humidity, 45 °C). Water was restricted during work to induce four different dehydration states: −1, −2, −3, and −4% body mass.	Short-term memory, arithmetic efficiency, and motor speed and attention deteriorated with increased dehydration, starting at 2% body mass loss.
Sharma et al., 1986 [64]	Investigate the effect of primary dehydration of various levels (1, 2 and 3% body-weight deficits) on mental functions in heat acclimatized subjects drawn from tropical regions of India	8 healthy men (age 21–24 y)	Crossover RCT	Subjects performed moderate work under hot and dry conditions (60% relative humidity, 45 °C) or hot and humid conditions (30% relative humidity, 45 °C) until they reached their target dehydration states: 0 (water replenished), −1, −2, and −3% body weight. Cognition tests were performed after 90 min rest in neutral conditions (27 °C, 50% relative humidity).	Symbol classification was not affected by dehydration. Concentration, memory, and perceptual motor coordination decreased at 2% and 3% body mass loss, compared to 0% dehydration.
**Headache**					
Spigt et al., 2012 [65]	Investigate the effects of increased water intake on headache	102 adults who had at least two episodes of moderately intense headache or at least five mildly intense episodes per month and a total fluid intake of less than 2.5 L/day. Control group: average age 45 y; water group: average age 47 y.	Parallel RCT	Both groups received written instructions about stress reduction and sleep improvement strategies. Group 1: Also instructed to consume an additional 1.5 L water/d (*n* = 52, 16/36 M/F). Group 2: No additional intervention (*n* = 50, 13/37 M/F). 3 month intervention.	Subjects who drank more water reported better migraine specific quality of life. 47% in the intervention (water) group self-reported improvement against 25% in controls. Drinking more water did not result in relevant changes in objective effect parameters, such as days with at least moderate headache or days with medication use
Spigt et al., 2005 [66]	Explore whether there could be a positive effect of increased water intake in headache patients	15 adults who frequently (once a week or more) suffered from migraine or tension-type headache, mean age 44 y	Parallel RCT	Group 1: Instructed to consume an additional 1.5 L water/d (*n* = 8). Group 2: Received placebo tablet (*n* = 7). 3 month intervention.	Additional water consumption decreased total number of hours of headache and headache intensity, but effects were not statistically significant.

Abbreviations: C, Celsius; d, day; EFSA, European Food Safety Authority; F, female; g, grams; h, hours; IOM, Institute of Medicine; kg, kilogram; L, liter; M, male; min, minute; mL, milliliter; MRI, magnetic reasonance imaging; *n*, sample size; oz, ounces; POMS, Profile of Mood States; RCT, randomized clinical trial; Uosm, urine osmolality; VAS, Visual Analogue Scale; y, years. ^1^ Intervention trials published since inception through April 2018.

**Table 3:** Intervention Studies on Hydration and Gastrointestinal Function ^1^.

Citation	Study Objective	Population	Design	Intervention	Summary/Conclusion
Anti et al., 1998 [73]	Determine the effects of a high-fiber diet and fluid supplementation in patients with functional chronic constipation	117 adults with chronic functional constipation (age 18–50 y). Baseline fluid intake: Group 1: 1.0 L (SD 0.2) and Group 2: 1.0 L (SD 0.4)	Parallel RCT	Group 1 (*n* = 58, 20/38 M/F) consumed standard diet providing 25 g fiber with ad libitum fluid intake. Group 2 (*n* = 59, 23/36 M/F) consumed standard diet providing 25 g fiber with 2 L/d fluid for 2 months	Fluid intake was greater in Group 2 (average 2.1 L/d) vs. Group 1 (average 1.1 L/d). Group 2 had greater increases in stool frequency and decreases in laxative use compared to Group 1.
Chung et al., 1999 [75]	Examine the effect of excess fluid (isotonic and hypotonic) on the actual stool output as measured by stool weight while simultaneously monitoring the urine output in 15 healthy volunteers	15 adults age 23 to 46 y. Baseline fluid intake: Group 1: 1.38 L (SD 0.93) and Group 2:1.20 L (SD 0.29).	Parallel	Group 1 (*n* = 9, 4/5 M/F): Additional intake of near isotonic fluid (Gatorade); Group 2 (*n* = 6, 3/3 M/F): Additional intake of hypotonic solution (water). Both groups consumed additional 1 L/d of fluid for 2 days, followed by additional 2 L/d of fluid for the next 2 days.	No change in total stool weight in both groups. Stool frequency was not reported.
Ziegenhagen et al., 1991 [76]	Compare the long-term effects of wheat bran alone vs. wheat bran with fluid addition on gastrointestinal function in healthy subjects	11 adults (55% F), age 19–33 y	Crossover RCT	Period 1: 15 g wheat bran twice/d. Period 2: 15 g wheat bran + 300 mL tea or water twice/d. Basal fluid intake restricted to 1–1.2 L/d. 14 d intervention, 7 d washout.	Gastric emptying was slower with bran vs. control and bran + fluid. Whole gut (oroanal) transit was shorter, while stool frequency and stool weight were greater with bran and bran + fluid vs. control. No effects due to addition of fluid were reported.
Klauser et al., 1990 [74]	Investigate whether fluid deprivation has an influence on colonic function	8 healthy men (age 21–28 y)	Crossover RCT	Control week: Consume >2500 mL fluid/d. Intervention week: Consume <500 mL fluid/d. 1 week intervention, 1 week washout.	Stool weight and frequency decreased with fluid restriction. No change in oroanal transit time.
van Nieuwenhoven et al., 2000	Examine the effect of dehydration on various gastrointestinal parameters during strenuous exercise.	10 healthy men (age 18–30 y)	Crossover RCT	Euhydration/control arm: Habitual fluid consumption. Dehydration arm: 15-min periods in a dry sauna interspersed with 10-min cooling off periods until 3% body mass loss was reached	Gastric emptying was significantly slower during dehydration. Orocecal transit time, intestinal permeability, and intestinal glucose absorption were unaffected by dehydration. Hydration status during euhydration/control arm was not assessed. Habitual fluid intake was not reported. (Only results from the pre-exercise/resting stage are reported herein).

Abbreviations: d, day; F, female; g, grams; L, liter; M, male; min, minute; mL, milliliter; *n*, sample size; RCT, randomized clinical trial; SD, standard deviation; y, years. ^1^ Intervention trials published since inception through April 2018.

**Table 4:** Intervention Studies on Hydration and Weight Management ^1^.

Citation	Study Objective	Population	Design	Intervention	Summary/Conclusion
Wong et al., 2017 [86]	Compare a standard weight-loss program with and without water	38 overweight and obese adolescents who reported drinking ≤4 cups of water/d; Control: 6M/13F, mean age 15.7 y; Water: 5M/14F, mean age 14.1 y	6 month parallel RCT	All participants received similar weight-reducing interventions (i.e., dietary counseling, daily text messages, and a cookbook with health guides). Control: No specific advice on water consumption. +Water: Received well-defined water messages through counseling and daily text messages, a water bottle, and a water pitcher with filters, and a target to increase habitual water intake to 8 cups/d.	Water group consumed more water [4.8 (3.8 to 5.9) cups of water/d] compared to the Control group [3.5 (2.6 to 4.4) cups/d]. Changes in BMI z-score and other anthropometric measures did not differ significantly between the two groups.
Parretti et al., 2015 [85]	Investigate the efficacy of water preloading before meals as a weight loss strategy for adults with obesity.	84 obese adults; Control: 15/28 M/F, mean age 57.8 y; Water: 15/26 M/F, mean age 55.1 y	12 week parallel RCT	All participants were given a face-to-face weight management consultation at baseline and a follow-up telephone consultation at 2 weeks. Control: Instructed to imagine their stomach was full before meals. +Water: Instructed to drink 500 mL of water 30 min before their main meals.	Water group lost 1.3 kg more than control group at 12 weeks.
Corney et al., 2015 [84]	Examine the effects of hydration status and/or fluid availability during eating on ad libitum energy intake	16 healthy males, average age 25 y.	Acute RCT	Subjects provided standard foods for 24 h which were designed so subjects are euhydrated or hypohydrated. Ad libitum breakfast was provided the next day.	Hydration status prior to ad libitum breakfast did not affect energy intake. Those who were hypohydrated (~1.8% body mass loss) consumed more fluids during breakfast compared to those who were euhydrated.

Abbreviations: BMI, body mass index; d, day; F, female; h, hours; kg, kilograms; M, male; mL, milliliter; *n*, sample size; RCT, randomized clinical trial; y, years. ^1^ Intervention trials published since January 2014 through April 2018; studies included in the 2018 Stookey review were not included in this table.

**Table 5:** Summary of Literature Findings.

Health Outcomes	Summary of Literature Findings
Skin Health	The effectiveness of additional water consumption on skin barrier function is unclear. A few studies suggest that increasing water consumption may improve the hydration of the stratum corneum layer of the epidermis, which plays a key role in skin barrier function. However, no changes to transepidermal water loss (measure of barrier integrity) were reported.
Cognition	Despite variability among study methodologies, dehydration impairs cognitive performance for tasks involving attention, executive function, and motor coordination when water deficits exceed 2% body mass loss. Cognitive domains involving lower order mental processing (e.g., simple reaction time) are less sensitive to changes in hydration status. In children, results from studies on hydration and cognition are mixed.
Mood and Fatigue	Hypohydration is associated with increased negative emotions such as anger, hostility, confusion, depression and tension as well as fatigue and tiredness. These findings are consistent in adults, but unclear and very limited in children.
Headache	The evidence is too limited to determine if hydration affects headache.
Kidney Stones	A significant association between high fluid intake and a lower risk of incident kidney stones has been reported, but data are limited.
Renal Function related to Toxin Elimination	There is not enough evidence to support commercial detox diets for toxin elimination.
Gastrointestinal Function and Constipation	Studies on hydration and general gastrointestinal function in healthy people are lacking. Clinical trials have been conducted on constipation, but currently do not support the use of increased fluid intake in the treatment of functional constipation. Further studies are necessary to understand the role of water and fluid consumption in the etiology and treatment of constipation.
Body Weight and Body Composition	Studies on fluid replacement of caloric beverages with non-caloric beverages have consistently resulted in lower energy intake. Existing data suggest that increased water consumption contributes to reductions in body fat and/or weight loss in obese adults, independent of changes in energy intake. Data in children are limited. More studies are needed to clarify the effect in both adults and children.

## References

[1] European Food Safety Authority (EFSA) Panel on Dietetic Products Nutrition and Allergies (NDA) (2010). Scientific Opinion on Dietary Reference Values for water. EFSA J..

[2] Food and Nutrition Board, Institute of Medicine (IOM) Panel on Dietary Reference Intakes for Electrolytes and Water (2005). Dietary Reference Intakes for Water, Potassium, Sodium, Chloride, and Sulfate.

[3] Cheuvront S.N., Kenefick R.W. (2014). Dehydration: Physiology, assessment, and performance effects. Compr. Physiol..

[4] Thomas D.R., Cote T.R., Lawhorne L., Levenson S.A., Rubenstein L.Z., Smith D.A., Stefanacci R.G., Tangalos E.G., Morley J.E., Dehydration Council (2008). Understanding clinical dehydration and its treatment. J. Am. Med. Dir. Assoc..

[5] Sawka M.N., Cheuvront S.N., Kenefick R.W. (2015). Hypohydration and Human Performance: Impact of Environment and Physiological Mechanisms. Sports Med..

[6] Bak A., Tsiami A., Greene C. (2017). Methods of Assessment of Hydration Status and their Usefulness in Detecting Dehydration in the Elderly. Curr. Res. Nutr. Food Sci..

[7] Cheuvront S.N., Ely B.R., Kenefick R.W., Sawka M.N. (2010). Biological variation and diagnostic accuracy of dehydration assessment markers. Am. J. Clin. Nutr..

[8] Cheuvront S.N., Kenefick R.W., Zambraski E.J. (2015). Spot Urine Concentrations Should Not be Used for Hydration Assessment: A Methodology Review. Int. J. Sport Nutr. Exerc. Metab..

[9] Armstrong L.E. (2005). Hydration assessment techniques. Nutr. Rev..

[10] Armstrong L.E. (2007). Assessing hydration status: The elusive gold standard. J. Am. Coll. Nutr..

[11] Cheuvront S.N. (2016). Urinalysis for hydration assessment: An age-old problem. Am. J. Clin. Nutr..

[12] Fortes M.B., Owen J.A., Raymond-Barker P., Bishop C., Elghenzai S., Oliver S.J., Walsh N.P. (2015). Is this elderly patient dehydrated? Diagnostic accuracy of hydration assessment using physical signs, urine, and saliva markers. J. Am. Med. Dir. Assoc..

[13] Heavens K.R., Charkoudian N., O’Brien C., Kenefick R.W., Cheuvront S.N. (2016). Noninvasive assessment of extracellular and intracellular dehydration in healthy humans using the resistance-reactance-score graph method. Am. J. Clin. Nutr..

[14] Hooper L., Bunn D., Jimoh F.O., Fairweather-Tait S.J. (2014). Water-loss dehydration and aging. Mech. Ageing Dev..

[15] Hooper L., Bunn D.K., Abdelhamid A., Gillings R., Jennings A., Maas K., Millar S., Twomlow E., Hunter P.R., Shepstone L. (2016). Water-loss (intracellular) dehydration assessed using urinary tests: How well do they work? Diagnostic accuracy in older people. Am. J. Clin. Nutr..

[16] Volkert D., Beck A.M., Cederholm T., Cruz-Jentoft A., Goisser S., Hooper L., Kiesswetter E., Maggio M., Raynaud-Simon A., Sieber C.C. (2018). ESPEN guideline on clinical nutrition and hydration in geriatrics. Clin. Nutr..

[17] Warren J., Guelinckx I., Livingstone B., Potischman N., Nelson M., Foster E., Holmes B. (2018). Challenges in the assessment of total fluid intake in children and adolescents: A discussion paper. Eur. J. Nutr..

[18] Gandy J. (2015). Water intake: Validity of population assessment and recommendations. Eur. J. Nutr..

[19] El-Sharkawy A.M., Sahota O., Lobo D.N. (2015). Acute and chronic effects of hydration status on health. Nutr. Rev..

[20] Kenefick R.W., Cheuvront S.N. (2012). Hydration for recreational sport and physical activity. Nutr. Rev..

[21] Manz F. (2013). Hydration and disease. J. Am. Coll. Nutr..

[22] Maughan R.J., Shirreffs S.M. (2010). Dehydration and rehydration in competitive sport. Scand. J. Med. Sci. Sports.

[23] Maughan R.J., Shirreffs S.M. (2010). Development of hydration strategies to optimize performance for athletes in high-intensity sports and in sports with repeated intense efforts. Scand. J. Med. Sci. Sports.

[24] Akdeniz M., Tomova-Simitchieva T., Dobos G., Blume-Peytavi U., Kottner J. (2018). Does dietary fluid intake affect skin hydration in healthy humans? A systematic literature review. Skin Res. Technol..

[25] Benton D., Young H.A. (2015). Do small differences in hydration status affect mood and mental performance?. Nutr. Rev..

[26] Masento N.A., Golightly M., Field D.T., Butler L.T., van Reekum C.M. (2014). Effects of hydration status on cognitive performance and mood. Br. J. Nutr..

[27] Cheungpasitporn W., Rossetti S., Friend K., Erickson S.B., Lieske J.C. (2016). Treatment effect, adherence, and safety of high fluid intake for the prevention of incident and recurrent kidney stones: A systematic review and meta-analysis. J. Nephrol..

[28] Boilesen S.N., Tahan S., Dias F.C., Melli L., de Morais M.B. (2017). Water and fluid intake in the prevention and treatment of functional constipation in children and adolescents: Is there evidence?. J. Pediatr..

[29] Stookey J.J. (2016). Negative, Null and Beneficial Effects of Drinking Water on Energy Intake, Energy Expenditure, Fat Oxidation and Weight Change in Randomized Trials: A Qualitative Review. Nutrients.

[30] Dabrowska A.K., Spano F., Derler S., Adlhart C., Spencer N.D., Rossi R.M. (2018). The relationship between skin function, barrier properties, and body-dependent factors. Skin Res. Technol..

[31] Kolarsick P.A.J., Kolarsick M.A., Goodwin C. (2011). Anatomy and Physiology of the Skin. J. Dermatol. Nurse Assoc..

[32] Rittie L., Fisher G.J. (2015). Natural and sun-induced aging of human skin. Cold Spring Harb. Perspect. Med..

[33] Palma L., Marques L.T., Bujan J., Rodrigues L.M. (2015). Dietary water affects human skin hydration and biomechanics. Clin. Cosmet. Investig. Dermatol..

[34] Palma M.L., Monteiro C., Bujan L.T.M.J., Rodrigues L.M. (2012). Relationship between the dietary intake of water and skin hydration. Biomed. Biopharm. Res..

[35] Palma M.L., Tavares L., Fluhr J.W., Bujan M.J., Rodrigues L.M. (2015). Positive impact of dietary water on in vivo epidermal water physiology. Skin Res. Technol..

[36] Mac-Mary S., Creidi P., Marsaut D., Courderot-Masuyer C., Cochet V., Gharbi T., Guidicelli-Arranz D., Tondu F., Humbert P. (2006). Assessment of effects of an additional dietary natural mineral water uptake on skin hydration in healthy subjects by dynamic barrier function measurements and clinic scoring. Skin Res. Technol..

[37] Williams S., Krueger N., Davids M., Kraus D., Kerscher M. (2007). Effect of fluid intake on skin physiology: Distinct differences between drinking mineral water and tap water. Int. J. Cosmet. Sci..

[38] Edmonds C.J., Crosbie L., Fatima F., Hussain M., Jacob N., Gardner M. (2017). Dose-response effects of water supplementation on cognitive performance and mood in children and adults. Appetite.

[39] Trinies V., Chard A.N., Mateo T., Freeman M.C. (2016). Effects of Water Provision and Hydration on Cognitive Function among Primary-School Pupils in Zambia: A Randomized Trial. PLoS ONE.

[40] Perry C.S., Rapinett G., Glaser N.S., Ghetti S. (2015). Hydration status moderates the effects of drinking water on children’s cognitive performance. Appetite.

[41] Booth P., Edmonds C.J. (2012). Water supplementation improves visual attention and fine motor skills in schoolchildren. Educ. Health.

[42] Fadda R., Rapinett G., Grathwohl D., Parisi M., Fanari R., Calo C.M., Schmitt J. (2012). Effects of drinking supplementary water at school on cognitive performance in children. Appetite.

[43] Kempton M.J., Ettinger U., Foster R., Williams S.C., Calvert G.A., Hampshire A., Zelaya F.O., O’Gorman R.L., McMorris T., Owen A.M. (2011). Dehydration affects brain structure and function in healthy adolescents. Hum. Brain Mapp..

[44] Edmonds C.J., Jeffes B. (2009). Does having a drink help you think? 6–7-Year-old children show improvements in cognitive performance from baseline to test after having a drink of water. Appetite.

[45] Edmonds C.J., Burford D. (2009). Should children drink more water?: The effects of drinking water on cognition in children. Appetite.

[46] Benton D., Burgess N. (2009). The effect of the consumption of water on the memory and attention of children. Appetite.

[47] Stachenfeld N.S., Leone C.A., Mitchell E.S., Freese E., Harkness L. (2018). Water intake reverses dehydration associated impaired executive function in healthy young women. Physiol. Behav..

[48] Benton D., Jenkins K.T., Watkins H.T., Young H.A. (2016). Minor degree of hypohydration adversely influences cognition: A mediator analysis. Am. J. Clin. Nutr..

[49] Pross N., Demazieres A., Girard N., Barnouin R., Metzger D., Klein A., Perrier E., Guelinckx I. (2014). Effects of changes in water intake on mood of high and low drinkers. PLoS ONE.

[50] Edmonds C.J., Crombie R., Ballieux H., Gardner M.R., Dawkins L. (2013). Water consumption, not expectancies about water consumption, affects cognitive performance in adults. Appetite.

[51] Lindseth P.D., Lindseth G.N., Petros T.V., Jensen W.C., Caspers J. (2013). Effects of hydration on cognitive function of pilots. Mil. Med..

[52] Edmonds C.J., Crombie R., Gardner M.R. (2013). Subjective thirst moderates changes in speed of responding associated with water consumption. Front. Hum. Neurosci..

[53] Ely B.R., Sollanek K.J., Cheuvront S.N., Lieberman H.R., Kenefick R.W. (2013). Hypohydration and acute thermal stress affect mood state but not cognition or dynamic postural balance. Eur. J. Appl. Physiol..

[54] Pross N., Demazieres A., Girard N., Barnouin R., Santoro F., Chevillotte E., Klein A., Le Bellego L. (2013). Influence of progressive fluid restriction on mood and physiological markers of dehydration in women. Br. J. Nutr..

[55] Armstrong L.E., Ganio M.S., Casa D.J., Lee E.C., McDermott B.P., Klau J.F., Jimenez L., Le Bellego L., Chevillotte E., Lieberman H.R. (2012). Mild dehydration affects mood in healthy young women. J. Nutr..

[56] Ganio M.S., Armstrong L.E., Casa D.J., McDermott B.P., Lee E.C., Yamamoto L.M., Marzano S., Lopez R.M., Jimenez L., Le Bellego L. (2011). Mild dehydration impairs cognitive performance and mood of men. Br. J. Nutr..

[57] Kempton M.J., Ettinger U., Schmechtig A., Winter E.M., Smith L., McMorris T., Wilkinson I.D., Williams S.C., Smith M.S. (2009). Effects of acute dehydration on brain morphology in healthy humans. Hum. Brain Mapp..

[58] Petri N.M., Dropulic N., Kardum G. (2006). Effects of voluntary fluid intake deprivation on mental and psychomotor performance. Croat. Med. J..

[59] Szinnai G., Schachinger H., Arnaud M.J., Linder L., Keller U. (2005). Effect of water deprivation on cognitive-motor performance in healthy men and women. Am. J. Physiol. Regul. Integr. Comp. Physiol..

[60] Shirreffs S.M., Merson S.J., Fraser S.M., Archer D.T. (2004). The effects of fluid restriction on hydration status and subjective feelings in man. Br. J. Nutr..

[61] Neave N., Scholey A.B., Emmett J.R., Moss M., Kennedy D.O., Wesnes K.A. (2001). Water ingestion improves subjective alertness, but has no effect on cognitive performance in dehydrated healthy young volunteers. Appetite.

[62] Rogers P.J., Kainth A., Smit H.J. (2001). A drink of water can improve or impair mental performance depending on small differences in thirst. Appetite.

[63] Gopinathan P.M., Pichan G., Sharma V.M. (1988). Role of dehydration in heat stress-induced variations in mental performance. Arch. Environ. Health.

[64] Sharma V.M., Sridharan K., Pichan G., Panwar M.R. (1986). Influence of heat-stress induced dehydration on mental functions. Ergonomics.

[65] Spigt M., Weerkamp N., Troost J., van Schayck C.P., Knottnerus J.A. (2012). A randomized trial on the effects of regular water intake in patients with recurrent headaches. Fam. Pract..

[66] Spigt M.G., Kuijper E.C., Schayck C.P., Troost J., Knipschild P.G., Linssen V.M., Knottnerus J.A. (2005). Increasing the daily water intake for the prophylactic treatment of headache: A pilot trial. Eur. J. Neurol..

[67] Alelign T., Petros B. (2018). Kidney Stone Disease: An Update on Current Concepts. Adv. Urol..

[68] Dion M., Ankawi G., Chew B., Paterson R., Sultan N., Hoddinott P., Razvi H. (2016). CUA guideline on the evaluation and medical management of the kidney stone patient—2016 update. Can. Urol. Assoc. J..

[69] Pearle M.S., Goldfarb D.S., Assimos D.G., Curhan G., Denu-Ciocca C.J., Matlaga B.R., Monga M., Penniston K.L., Preminger G.M., Turk T.M. (2014). Medical management of kidney stones: AUA guideline. J. Urol..

[70] Borghi L., Meschi T., Amato F., Briganti A., Novarini A., Giannini A. (1996). Urinary volume, water and recurrences in idiopathic calcium nephrolithiasis: A 5-year randomized prospective study. J. Urol..

[71] Sarica K., Inal Y., Erturhan S., Yagci F. (2006). The effect of calcium channel blockers on stone regrowth and recurrence after shock wave lithotripsy. Urol. Res..

[72] Leiper J.B. (2015). Fate of ingested fluids: Factors affecting gastric emptying and intestinal absorption of beverages in humans. Nutr. Rev..

[73] Anti M., Pignataro G., Armuzzi A., Valenti A., Iascone E., Marmo R., Lamazza A., Pretaroli A.R., Pace V., Leo P. (1998). Water supplementation enhances the effect of high-fiber diet on stool frequency and laxative consumption in adult patients with functional constipation. Hepatogastroenterology.

[74] Klauser A.G., Beck A., Schindlbeck N.E., Muller-Lissner S.A. (1990). Low fluid intake lowers stool output in healthy male volunteers. Z. Gastroenterol..

[75] Chung B.D., Parekh U., Sellin J.H. (1999). Effect of increased fluid intake on stool output in normal healthy volunteers. J. Clin. Gastroenterol..

[76] Ziegenhagen D.J., Tewinkel G., Kruis W., Herrmann F. (1991). Adding more fluid to wheat bran has no significant effects on intestinal functions of healthy subjects. J. Clin. Gastroenterol..

[77] van Nieuwenhoven M.A., Vriens B.E., Brummer R.J., Brouns F. (2000). Effect of dehydration on gastrointestinal function at rest and during exercise in humans. Eur. J. Appl. Physiol..

[78] Akers J.D., Cornett R.A., Savla J.S., Davy K.P., Davy B.M. (2012). Daily self-monitoring of body weight, step count, fruit/vegetable intake, and water consumption: A feasible and effective long-term weight loss maintenance approach. J. Acad. Nutr. Diet..

[79] Dennis E.A., Dengo A.L., Comber D.L., Flack K.D., Savla J., Davy K.P., Davy B.M. (2010). Water consumption increases weight loss during a hypocaloric diet intervention in middle-aged and older adults. Obesity.

[80] Muckelbauer R., Libuda L., Clausen K., Toschke A.M., Reinehr T., Kersting M. (2009). Promotion and provision of drinking water in schools for overweight prevention: Randomized, controlled cluster trial. Pediatrics.

[81] Stookey J.D., Del Toro R., Hamer J., Medina A., Higa A., Ng V., TinajeroDeck L., Juarez L. (2014). Qualitative and/or quantitative drinking water recommendations for pediatric obesity treatment. J. Obes. Weight Loss Ther..

[82] Hernandez-Cordero S., Popkin B.M. (2015). Impact of a Water Intervention on Sugar-Sweetened Beverage Intake Substitution by Water: A Clinical Trial in Overweight and Obese Mexican Women. Ann. Nutr. Metab..

[83] Hernandez-Cordero S., Barquera S., Rodriguez-Ramirez S., Villanueva-Borbolla M.A., Gonzalez de Cossio T., Dommarco J.R., Popkin B. (2014). Substituting water for sugar-sweetened beverages reduces circulating triglycerides and the prevalence of metabolic syndrome in obese but not in overweight Mexican women in a randomized controlled trial. J. Nutr..

[84] Corney R.A., Horina A., Sunderland C., James L.J. (2015). Effect of hydration status and fluid availability on ad-libitum energy intake of a semi-solid breakfast. Appetite.

[85] Parretti H.M., Aveyard P., Blannin A., Clifford S.J., Coleman S.J., Roalfe A., Daley A.J. (2015). Efficacy of water preloading before main meals as a strategy for weight loss in primary care patients with obesity: RCT. Obesity.

[86] Wong J.M.W., Ebbeling C.B., Robinson L., Feldman H.A., Ludwig D.S. (2017). Effects of Advice to Drink 8 Cups of Water per Day in Adolescents With Overweight or Obesity: A Randomized Clinical Trial. JAMA Pediatr..

[87] Chang T., Ravi N., Plegue M.A., Sonneville K.R., Davis M.M. (2016). Inadequate Hydration, BMI, and Obesity Among US Adults: NHANES 2009–2012. Ann. Fam. Med..

[88] Kenney E.L., Long M.W., Cradock A.L., Gortmaker S.L. (2015). Prevalence of Inadequate Hydration among US Children and Disparities by Gender and Race/Ethnicity: National Health and Nutrition Examination Survey, 2009–2012. Am. J. Public Health.

[89] Silva C.L., Topgaard D., Kocherbitov V., Sousa J.J., Pais A.A., Sparr E. (2007). Stratum corneum hydration: Phase transformations and mobility in stratum corneum, extracted lipids and isolated corneocytes. Biochim. Biophys. Acta.

[90] Wittbrodt M.T., Millard-Stafford M. (2018). Dehydration Impairs Cognitive Performance: A Meta-analysis. Med. Sci. Sports Exerc..

[91] Cunningham P., Noble H., Al-Modhefer A.K., Walsh I. (2016). Kidney stones: Pathophysiology, diagnosis and management. Br. J. Nurs..

[92] Masereeuw R., Russel F.G. (2001). Mechanisms and clinical implications of renal drug excretion. Drug Metab. Rev..

[93] Klein A.V., Kiat H. (2015). Detox diets for toxin elimination and weight management: A critical review of the evidence. J. Hum. Nutr. Diet..

[94] Khan L. (2018). Constipation Management in Pediatric Primary Care. Pediatr. Ann..

[95] Sharma A., Rao S. (2017). Constipation: Pathophysiology and Current Therapeutic Approaches. Handb. Exp. Pharmacol..

[96] Thornton S.N. (2016). Increased Hydration Can Be Associated with Weight Loss. Front. Nutr..

[97] Drewnowski A., Rehm C.D., Constant F. (2013). Water and beverage consumption among adults in the United States: Cross-sectional study using data from NHANES 2005-2010. BMC Public Health.

[98] Stachenfeld N.S. (2008). Sex hormone effects on body fluid regulation. Exerc. Sport Sci. Rev..

[99] Stachenfeld N.S. (2014). Hormonal changes during menopause and the impact on fluid regulation. Reprod. Sci..

[100] Stookey J.D. (2005). High prevalence of plasma hypertonicity among community-dwelling older adults: Results from NHANES III. J. Am. Diet. Assoc..

[101] Maughan R.J. (2012). Hydration, morbidity, and mortality in vulnerable populations. Nutr. Rev..

[102] Stotts N.A., Hopf H.W. (2003). The link between tissue oxygen and hydration in nursing home residents with pressure ulcers: Preliminary data. J. Wound Ostomy Cont. Nurs..

